# Como Incorporar o Sistema de Detecção Dielétrica Remota e o Ultrassom Pulmonar em Pacientes com Insuficiência Cardíaca Congestiva Aguda

**DOI:** 10.36660/abc.20250053

**Published:** 2025-04-23

**Authors:** Toshihide Izumida, Teruhiko Imamura

**Affiliations:** 1 University of Toyama Toyama Japão University of Toyama, Toyama – Japão

**Keywords:** Insuficiência Cardíaca, Hemodinâmica, Pulmão

**Ao Editor**

A avaliação precisa da congestão pulmonar é essencial para o gerenciamento ideal da insuficiência cardíaca congestiva. O sistema de sensoriamento dielétrico remoto (ReDS) representa uma tecnologia não invasiva desenvolvida recentemente, projetada para quantificar o volume de fluido pulmonar sem exigir conhecimento especializado. Kobalava et al. demonstraram uma correlação moderada entre o sistema ReDS e o ultrassom pulmonar.^1^ No entanto, várias preocupações justificam uma discussão mais aprofundada.

No estudo atual, certos pacientes apresentaram achados mais elevados na ultrassonografia pulmonar (conforme indicado pela soma das linhas B), apesar dos valores mais baixos de ReDS (%).^1^ Os autores poderiam fornecer uma explicação para essa discordância observada? Vale a pena notar que vários fatores de confusão podem influenciar as medições de ReDS (%). Por exemplo, ReDS (%) tende a ser subestimado em indivíduos com doença pulmonar obstrutiva crônica ou baixo índice de massa corporal.^2^

Dada a variabilidade interindividual nas medições de ReDS (%), comparações diretas de valores absolutos de ReDS (%) entre pacientes podem representar desafios significativos. Como alternativa, a razão de ReDS (%) entre admissão hospitalar e alta pode servir como uma métrica valiosa para avaliar a melhora da congestão pulmonar durante a hospitalização índice.^3^

A definição de congestão pulmonar significativa é geralmente baseada em uma soma de linhas B igual ou superior a três, em vez de cinco.^4^ Como a concordância entre o sistema ReDS e o ultrassom pulmonar seria afetada se esse limite revisado para congestão pulmonar fosse aplicado?

Com base em suas descobertas, como o sistema ReDS e o ultrassom pulmonar podem ser integrados de forma ideal à prática clínica? Por exemplo, sugerimos utilizar o sistema ReDS para coortes de pacientes menos criticamente doentes, pois ele é particularmente eficaz na identificação de congestão pulmonar subclínica.^4^ Por outro lado, o ultrassom pulmonar pode ser mais apropriado para pacientes gravemente enfermos, pois esses indivíduos geralmente enfrentam desafios para assumir uma posição sentada com respiração natural, o que é necessário para a aplicação e medição adequadas do dispositivo ReDS.^2^ O ultrassom pulmonar pode ser aplicado sem a cooperação do paciente.
