# Supplementary material for: Como Incorporar o Sistema de Detecção Dielétrica Remota e o Ultrassom Pulmonar em Pacientes com Insuficiência Cardíaca Congestiva Aguda
Source: Arq Bras Cardiol. 2025 Apr 23;122(4):e20250053. [Article in Portuguese] doi: 10.36660/abc.20250053 (PMC12094292; doi:10.36660/abc.20250053)
Supplement: Translation

# Translation: How to Incorporate Remote Dielectric Sensing System and Lung Ultrasound in Patients with Acute Congestive Heart Failure

**DOI:** 10.36660/abc.20250053i

**Authors:** Toshihide Izumida, Teruhiko Imamura

**Affiliations:** 1 Toyama Japan University of Toyama, Toyama – Japan

**Keywords:** Heart Failure, Hemodynamics, Lung

**To Editor**

Accurate assessment of pulmonary congestion is pivotal for the optimal management of congestive heart failure. The remote dielectric sensing (ReDS) system represents a recently developed non-invasive technology designed to quantify lung fluid volume without requiring specialized expertise. Kobalava et al. demonstrated a moderate correlation between the ReDS system and lung ultrasound.^1^ However, several concerns warrant further discussion.

In the current study, certain patients exhibited higher lung ultrasound findings (as indicated by the sum of B-lines) despite lower ReDS (%) values.^1^ Could the authors provide an explanation for this observed discordance? It is worth noting that various confounding factors may influence ReDS (%) measurements. For example, ReDS (%) tends to be underestimated in individuals with chronic obstructive pulmonary disease or low body mass index.^2^

Given the inter-individual variability in ReDS (%) measurements, direct comparisons of absolute ReDS (%) values across patients may pose significant challenges. As an alternative, the ratio of ReDS (%) between hospital admission and discharge could serve as a valuable metric to evaluate the improvement of pulmonary congestion during the index hospitalization.^3^

The definition of significant pulmonary congestion is generally based on a sum of B-lines equal to or exceeding three rather than five.^4^ How would the concordance between the ReDS system and lung ultrasound be affected if this revised threshold for pulmonary congestion were applied?

Building upon their findings, how might the ReDS system and lung ultrasound be optimally integrated into clinical practice? For instance, we suggest utilizing the ReDS system for less critically ill patient cohorts, as it is particularly effective in identifying subclinical pulmonary congestion.^4^ Conversely, lung ultrasound may be more appropriate for critically ill patients, as these individuals often face challenges in assuming a seated position with natural breathing, which is required for proper ReDS device application and measurement.^2^ The lung ultrasound can be applied without patients’ cooperation.
