# Supplementary material for: Como Incorporar o Sistema de Detecção Dielétrica Remota e o Ultrassom Pulmonar em Pacientes com Insuficiência Cardíaca Congestiva Aguda
Source: Arq Bras Cardiol. 2025 Apr 23;122(4):e20250053. [Article in Portuguese] doi: 10.36660/abc.20250053 (PMC12094292; doi:10.36660/abc.20250053)
Supplement: Related texts

# Reply: Carta-resposta

**Authors:** Zhanna Kobalava, Ayten Fuadovna Safarova, Veronika Tolkacheva, Flora Elisa Cabello Montoya, Olga Tairovna Zorya, Ivan Sergeevich Nazarov, Artem Alekseevich Lapshin, Ilya Pavlovich Smirnov, Nutsiko Ivanovna Khutsishvili, Maria Vatsik-Gorodetskaya

**Affiliations:** 1 RUDN University Moskva Federação Russa RUDN University, Moskva, Moskovskaa oblast’ – Federação Russa

Uma proporção significativa de pacientes apresentou um resultado desfavorável na gravidade da congestão ao utilizar ultrassom pulmonar e ReDS. A congestão foi detectada com mais frequência pelo ReDS do que pelo ultrassom, no entanto, houve observações isoladas com resultados opostos. Infelizmente, dada a limitação da nossa amostra, não seremos capazes de fornecer diferenças estatisticamente confirmadas entre grupos de pacientes com um desfecho desfavorável e grupos com desfechos semelhantes. No entanto, com base em dados da literatura e em nossa própria experiência clínica, podemos dizer que algumas características dos pacientes podem ter o efeito oposto nos dados obtidos pelo ReDS e pelo ultrassom. Assim, a obesidade subestimará o número de linhas B¹ e superestimará o valor do ReDS² e vice-versa, com um peso corporal mais baixo. Em pacientes com DPOC/BA, o número de linhas B pode ser também paradoxalmente alto, mesmo na ausência de insuficiência cardíaca,³ e, de acordo com o ReDS, a congestão nesses pacientes pode não ser acentuada.^4^ Embora seja mais frequentemente representada por observações individuais, em nosso trabalho também observamos uma congestão estatisticamente significativamente mais baixa em pacientes com DPOC concomitante. Também observamos uma correlação negativa do ReDS com a idade, na ausência de relação entre idade e o número de linhas B. Curiosamente, o maior número de resultados discordantes foi na alta, o que provavelmente reflete a taxa diferente de eliminação da congestão em diferentes compartimentos (vascular, intersticial, etc.).

A dinâmica do ReDS durante a hospitalização pode ser avaliada por diferentes métodos, e a razão entre o valor do ReDS na alta e na admissão^5^ pode ter um valor prognóstico maior do que a presença/ausência de congestão na alta, determinada por valores padrão. No entanto, o valor preditivo do ReDS não foi avaliado no âmbito do trabalho publicado. Deve-se observar que o valor da razão proposto de >100% reflete apenas a ausência de dinâmica positiva (ou dinâmica negativa). Deve-se ressaltar que a maioria dos pacientes terá dinâmica positiva; para eles, a conclusão sobre a razão <100% é irrelevante, sendo que é a presença de congestão residual na alta que exige avaliação nesses pacientes para entender as táticas e os riscos subsequentes. Além disso, essa razão pode ser interpretada de forma equivocada em pacientes com insuficiência ventricular direita.

## References

[1] Brainin P, Claggett B, Lewis EF, Dwyer KH, Merz AA, Silverman MB, Swamy V, Biering-Sørensen T, Rivero J, Cheng S, McMurray JJV, Solomon SD, Platz E (2020). Body mass index and B-lines on lung ultrasonography in chronic and acute heart failure. ESC Heart Fail.

[2] Izumida T, Imamura T, Nakagaito M, Onoda H, Tanaka S, Ushijima R, Fujioka H, Kakeshita K, Kinugawa K (2023). Association Between Remote Dielectric Sensing and Body Mass Index. Int Heart J.

[3] Lajili F, Toumia M, Sekma A, Bel Haj Ali K, Sassi S, Zorgati A, Yaakoubi H, Youssef R, Grissa MH, Beltaief K, Mezgar Z, Khrouf M, Chamtouri I, Bouida W, Boubaker H, Msolli MA, Dridi Z, Boukef R, Nouira S (2024). Value of Lung Ultrasound Sonography B-Lines Quantification as a Marker of Heart Failure in COPD Exacerbation. Int J Chron Obstruct Pulmon Dis.

[4] Izumida T, Imamura T, Tanaka S, Kinugawa K (2022). Experience with remote dielectric sensing (ReDS) for acute decompensated heart failure complicated by chronic obstructive pulmonary disease. J Cardiol Cases.

[5] Izumida T, Imamura T, Koi T, Nakagaito M, Onoda H, Tanaka S, Ushijima R, Kataoka N, Nakamura M, Sobajima M, Fukuda N, Ueno H, Kinugawa K (2024). Prognostic impact of residual pulmonary congestion assessed by remote dielectric sensing system in patients admitted for heart failure. ESC Heart Fail.

# Reply

**Authors:** Zhanna Kobalava, Ayten Fuadovna Safarova, Veronika Tolkacheva, Flora Elisa Cabello Montoya, Olga Tairovna Zorya, Ivan Sergeevich Nazarov, Artem Alekseevich Lapshin, Ilya Pavlovich Smirnov, Nutsiko Ivanovna Khutsishvili, Maria Vatsik-Gorodetskaya

**Affiliations:** 1 RUDN University Moskva Russia RUDN University, Moskva, Moskovskaa oblast’ – Russia

A significant proportion of patients had an unfavorable result in the severity of congestion when using lung ultrasound and ReDS. Congestion was more often detected by ReDS than by ultrasound, however, there were isolated observations with opposite results. Unfortunately, given the limitations of our sample, we will not be able to provide statistically confirmed differences between groups of patients with a discounted outcome and groups with matching outcomes. However, based on literature data and our own clinical experience, we can say that some characteristics of patients may have the opposite effect on the obtained ReDS and ultrasound data. Thus, obesity will underestimate the number of B-lines^1^ and overestimate the value of ReDS^2^ and vice versa with a lower body weight. In patients with COPD/BA, the number of B-lines may also be paradoxically high even in the absence of heart failure,^3^ and according to ReDS, congestion in such patients may be not marked.^4^ Although it is more often represented by individual observations, in our work we also noted a statistically significantly lower congestion in patients with concomitant COPD. We also noted a negative correlation of ReDS with age, in the absence of a relationship between age and the number of B-lines. Interestingly, the greatest number of discordant results was at discharge, which probably reflects the different rate of elimination of congestion in different compartments (vascular, interstitial, etc.).

The dynamics of ReDS during hospitalization can be assessed by different methods, and the ratio of the ReDS value at discharge and admission^5^ may have a prognostic value greater than the presence/absence of congestion at discharge, determined by standard values. However, the predictive value of ReDS was not evaluated in the framework of the published work. It should be noted that the proposed ratio value of >100% only reflects the absence of positive dynamics (or negative dynamics). It should be noted that most patients will have positive dynamics, for them the conclusion about the ratio <100% is meaningless, it is the presence of residual congestion at discharge that requires assessment in such patients to understand further tactics and risks. In addition, this ratio may be misinterpreted in patients with right ventricular failure.
